# Latitudinal Pigmentation Variation Contradicts Ultraviolet Radiation Exposure: A Case Study in Tropical Indian *Drosophila melanogaster*

**DOI:** 10.3389/fphys.2019.00084

**Published:** 2019-02-11

**Authors:** Subhash Rajpurohit, Paul S. Schmidt

**Affiliations:** ^1^Division of Biological and Life Sciences, School of Arts and Sciences, Ahmedabad University, Ahmedabad, India; ^2^Department of Biology, University of Pennsylvania, Philadelphia, PA, United States

**Keywords:** pigmentation, geographical variations, ultraviolet light, *Drosophila melanogaster*, Indian subcontinent

## Abstract

The effects of ultraviolet radiation (UV) on the animal body have been reported in many studies, and melanin has emerged as a protective mechanism. In smaller insects such as *Drosophila,* replicated patterns of geographical variation in pigmentation have been observed on multiple continents. Such patterns are particularly pronounced on the Indian subcontinent where several species show a parallel cline in pigmentation traits. However, the potential role of UV exposure in generating the observed patterns of pigmentation variation has not been addressed. Here, we examine the association between UV intensity and body pigmentation in *D. melanogaster* natural populations collected along the latitudinal gradient of the Indian subcontinent. A strong negative relationship was observed between UV intensity and body pigmentation. This analysis clearly indicates that, in the sampled populations, pigmentation variation is independent of UV exposure and related selection pressures. Patterns of pigmentation in natural populations from the Indian subcontinent are better predicted by latitude itself and temperature-related climatic variables.

## Introduction

The outermost coverings of animal bodies are equipped with variety of defense mechanisms, such as the vertebrate skin with spines or scales, as well as the hard cuticle with epicuticular lipids, melanin and associated pigments in a variety of invertebrates. The biochemical properties of melanin molecules are complicated and poorly studied with respect to their potential adaptive roles in wild populations. These molecules are an integral component of the insect cuticle and are considered to be targets of selection both within and among the phenotypic divergence of species (Wittkopp and Beldade, 2009).

Pigmentation variation in *Drosophila* species has been correlated with cuticle strength, fertility, desiccation tolerance, vision, circadian activity, mating and UV resistance (Kalmus, 1941; Fahmy and Fahmy, 1959; Jacobs, 1985; Newby and Jackson, 1991; Borycz et al., 2002; Brisson et al., 2005; Dev et al., 2013; Bastide et al., 2014). In addition to inter-specific patterns in pigmentation, there is also extensive variation within species (Rajpurohit et al., 2008; Parkash et al., 2009a, 2010; Singh et al., 2009). Traits showing greater variability across geography are commonly associated with the climatic variables (Rajpurohit and Nedved, 2013). It has been suggested that pigmentation variations are associated with temperature regime and UV intensity, which change in a predictive direction as a function of latitude (Ziemke et al., 2000; Rajpurohit et al., 2008).

In the literature, the effects of UV radiation on smaller insects like *Drosophila* have rarely been addressed. Furthermore, the majority of the associated data and findings come from laboratory experimentation (Jacobs, 1985; Mosse and Lyakh, 1994). In other vertebrates including humans, correlations between skin tone and UV intensity have been documented on a geographical scale (Jablonski and Chaplin, 2010). In insects, the associations between intraspecific variation in pigmentation and geographic variation in UV have rarely been addressed, other than two recent studies on *Drosophila* species from Africa and islands off the coast of the Gulf of Guinea (Matute and Harris, 2013; Bastide et al., 2014). Unfortunately, pigmentation and UV interactions have not been studied in Indian drosophilids where several species show parallel latitudinal cline in pigmentation (Rajpurohit and Nedved, 2013; Kalra et al., 2014).

Here, we utilize natural populations of *Drosophila melanogaster* collected along the latitudinal gradient on the Indian subcontinent (range of > 2000 km) to examine the association between UV intensity and pigmentation pattern. We performed regression analysis of pigmentation with geographical and climatic variables, including UV intensity, and tested the “*melanism-UV*” hypothesis and predicted a positive correlation between UV intensity and pigmentation variation along the Indian latitudes.

## Materials and Methods

**FIGURE 1:**
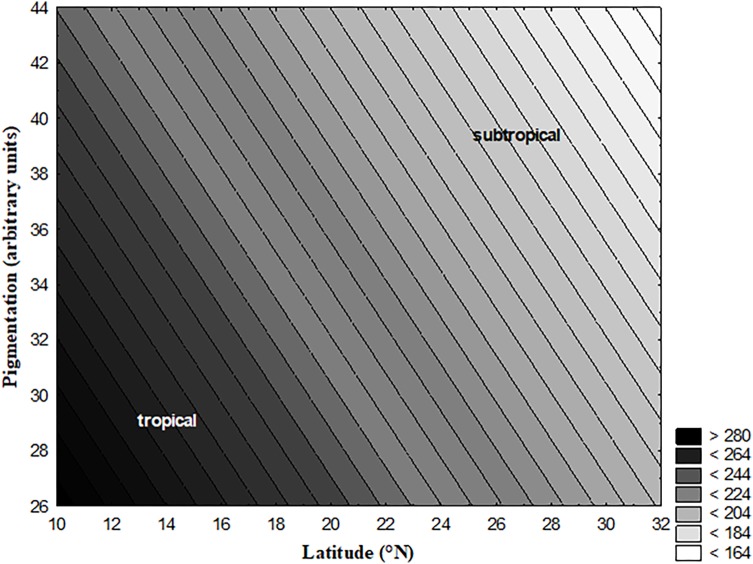
3D Contour plot showing erythemal ultraviolet data against latitude and pigmentation. The data presented in this figure has been developed at NASA Goddard Space Flight Center (GSFC). The erythemal ultraviolet values presented here in this figure are averages of monthly values recorded from 1996 to 2003. The values shown on *Z*-axis (UV Range) were calculated from mantissa (M) and exponent (E). For further details see ftp://toms.gsfc.nasa.gov/pub/eptoms/data/erythemal/Y2005/L3_eryln_ept_20051214.txt.

Abdominal pigmentation data shown in Table 1 was obtained with permission from Rajpurohit et al. (2007); *Drosophila Information Service* 90:70–79. The data is presented as the sum of all seven abdominal tergites of female *D. melanogaster*. For abdominal pigmentation quantification, the method described by David et al. (1990) was used where degree of pigmentation was estimated from a lateral view of the female abdomen giving values ranging from 0 (no pigment) up to 10 (completely pigmented) for each of the seven segments (see Figure 1; David et al., 1990). This study was conducted at 25°C temperature. For further details see David et al. (1990) and Rajpurohit et al. (2007).

**Table 1:** Geographical and climatic data for the five locations of the origin of the populations of *D. melanogaster* from the Indian subcontinent.

Geographical locations	Latitude (°N)	Longitude (°E)	Altitude (m a.s.l.)	T_ave_ (°c)	T_cv_ (%)	UV Light (mW/m2)	Pigmentation (sum)
Calicut	11.15 **(11.50)**	75.49 **(75.62)**	10	27.5	5.53	281.19	07.80
Ratnagiri	16.59 **(16.50)**	73.20 **(73.12)**	67	26.9	4.73	232.28	17.01
Deesa	24.12 **(24.50)**	72.12 **(71.87)**	136	26.7	18.18	218.79	24.00
Bareilly	28.22 **(28.50)**	79.42 **(79.37)**	173	25.2	25.85	183.88	24.40
Chandigarh	30.44 **(30.50)**	76.53 **(76.87)**	347	23.4	28.11	173.30	25.90


*UV data corresponds to the latitudes and longitudes showing in the brackets (in bold letters). These values are the closest to the NASA Goddard Space Flight Center (GSFC) data collection coordinates. T_ave_, Temperature average; T_cv_, coefficient of variance of temperature. Pigmentation data was used with permission from Rajpurohit et al. (2007).*

Climatic data for the sites of origin of populations were obtained from the climatological tables of the India Meteorological Department, and represent averages over the last 30 years. T_cv_ (coefficient of variance of temperature) data was calculated using monthly T_max_ and T_min_ for 12 months (for the 30 year time interval, 1960–1990).

Erythemal UV data was obtained from NASA Goddard Space Flight Center (GSFC^1^). The Erythemal Exposure data product is an estimate of the daily integrated ultraviolet irradiance calculated using a model for the susceptibility of caucasian skin to sunburn (erythema). This can be interpreted as an index of the potential for biological damage due to solar irradiation, given the column ozone amount and cloud conditions, as determined from measurements made with the Total Ozone Mapping Spectrometer (TOMS), on each day. The solar irradiances used in the calculation have units of nW mˆ{-2} nmˆ{-1} (nanowatts per square meter per nanometer; the “square meter” refers to the area on a horizontal surface, the “nanometer” refers to the wavelength of the light). Thus, after integrating over wavelength and time, one is left with units of J mˆ{-2} (Joules per square meter).

Ultraviolet radiation (UVR) is at shorter wavelengths than the visible spectrum (400 to 700 nm) and is divided into three components: UV-A (315 to 400 nm), UV-B (280 to 315 nm) and UV-C (less than 280 nm). The shorter wavelengths that comprise UV-B are the most dangerous portion of UV radiation that can reach ground level. Atmospheric ozone shields life at the surface from most of the UV-B and almost all of the UV-C. UV-A and UV-B are reduced by a small amount from Rayleigh scattering in the atmosphere. We used annual mean UV index and latitudinal data for each location as surrogate estimates of UVR exposure to facilitate the evaluation of hazardous incidences in relation to UVR exposure and geographic locations. Geographic latitude of residence is a routinely used proxy measure for UVR exposure.

We handled the statistical methods using averages of climatic parameters and population’s means of pigmentation. Regression analyses were carried out in exploratory analyses to examine associations between population trait means and the geographical and climatic variables. Under strong climatic selection, we might expect associations between trait means and climatic variables regardless of the distance between populations. Regression analysis is ideal in cases where we want to understand which among the independent variables are related to the dependent variable, and to explore the forms of these relationships. The analysis was performed in STATISTICA, version 7 (StatSoft).

## Results and Discussion

We found a positive correlation between latitude of the origin of populations and the degree of abdominal pigmentation; however, UV intensity was negatively correlated with the degree of pigmentation (Figure 1). The intensity of UV was higher toward lower latitudes/or tropical locations than higher latitudes/or subtropical locations (Figure 1). Neither pigmentation nor UV intensity was associated with longitude (Table 1). Latitudinal variations in pigmentation have been described from India in various species (Parkash et al., 2009b, 2010; Kalra et al., 2014). On the Indian subcontinent pigmentation increases toward the north, i.e., southern populations are significantly lighter in body pigmentation than northern populations. Researchers observed these variations in populations simultaneously under common garden conditions; these changes reflect genetic differences among populations. Based on populations mean values for body melanization (in percentages), correlations between melanization and various climatic parameters were obtained. Simple regression analysis of pigmentation with various geographical and climatic components revealed various responses. Latitude, altitude and T_cv_ were found to be positively associated with pigmentation whereas T_ave_ and UV intensity showed a negative relation to pigmentation (Table 2). Contrary to our initial assumption, here in this study pigmentation and UV intensity showed opposite trends along the latitudinal gradient of Indian subcontinent.

**Table 2:** Regression analysis of pigmentation with various geographical and climatic components.

Parameters	*r*	*b*	*p*
Latitude	0.96	0.89 ± 0.15	**0.009**
Longitude	0.19	0.50 ± 1.48	0.756
Altitude	0.81	0.04 ± 0.02	0.092
T_ave_	-0.74	-3.39 ± 1.76	0.149
T_cv_	0.85	0.58 ± 0.20	0.061
UV	-0.94	-0.16 ± 0.03	**0.015**


*This analysis is based on the data presented in Table 1. P < 0.05 is significant (shown in bold letters).*

Pigmentation is associated with various biological phenomena such as mimicry, camouflage, deflection, intraspecific recognition, mate choice, and in physiological processes such as thermoregulation, desiccation resistance and photo-protection (see True, 2003; Wittkopp and Beldade, 2009; Kronforst et al., 2012). Particularly, the involvement of pigmentation in photo-protection in *Drosophila* and other smaller size insects has not been well addressed under natural conditions. A handful of studies on laboratory adapted strains/ or mutants have been performed that focus on deleterious effects of ultraviolet light. For example, the *ebony* mutants of *D. melanogaster*, which are darker than wild type, have weaker wing cuticle and lower UV tolerance (Jacobs, 1985). Mosse and Lyakh (1994) exposed *Drosophila* populations to UV radiation for 115 generations and demonstrated that the populations exposed to long-term irradiation have the greatest number of mutations, thereby decreasing viability. Under natural conditions in the *D. cardini* species group, darker morphs are more prevalent in open habitats (exposed to direct sunlight and UVR) whereas lighter flies predominate in shaded habitats. Usually tropical locations are characterized by greater exposure to UV than temperate locations. However, *D. melanogaster* populations from tropical India are lighter in pigmentation than temperate French populations (Gibert et al., 1998), suggesting the association between UV intensity and latitude may be variable.

Parallel clines for body pigmentation have been reported in multiple *Drosophila* species from the Indian subcontinent (Rajpurohit et al., 2013). Among Indian populations of *D. melanogaster*, the observed pigmentation cline does not support the photo-protective involvement of this trait at the population level: the association between UV intensity and latitude exhibits the opposite pattern as the observed cline in pigmentation. Interestingly, Wittkopp et al. (2011) found a longitudinal cline in body pigmentation in *D. americana* in populations derived from the United States. However, UV intensity varies less as a function of longitude than it does with latitude (except in the case of elevation changes; Bastide et al., 2014) as UV intensities are higher close to the equator. Our findings are opposite to the prediction and to the results of Bastide et al. (2014). Matute and Harris (2013), assessed experimentally resistance to UV on *D. yakuba* which is polymorphic for abdominal pigmentation. They found that lighter flies resist better to UV. According to our data, melanin do not seem to be protective against UV.

The insect cuticle is a complex biological structure. Thus, selection may act on a variety of genes in the pigmentation pathways (Kronforst et al., 2012). Furthermore, at an ecological level pigmentation in the genus *Drosophila* is highly variable. It shows spectacular differences between species, between populations of the same species, and within a population (Hollocher et al., 2000; Rajpurohit et al., 2008; Parkash et al., 2009a). This suggests that the exploitation of a wide range of microhabitats by drosophilids is associated with variation and divergence in pigmentation pattern, potentially due to the pleiotropic fitness effects of pigmentation pattern across a range of spatiotemporal scales. However, these complex molecular mechanisms have not been tested experimentally under laboratory conditions using recently collected out-bred populations exhibiting variation in pigmentation. Finally, this suggests an opportunity for future work to rigorously test the *melanism-UV* hypothesis.

## Conclusion

We performed a climatic regression analysis of *D. melanogaster* female pigmentation with geographical and climatic variables along the Indian latitudes. The strongest pattern we observed was the association between latitude and UV intensity. However, pigmentation increases positively with increasing latitude whereas UV intensity decreases with increasing latitude. Therefore UV intensity and pigmentation variation do not exhibit parallel trends along the Indian latitudes. Latitude explained most of the variation for the observed pigmentation in *D. melanogaster* on the Indian subcontinent, suggesting that UV exposure alone is insufficient to explain the widespread variation in pigmentation in natural populations.

## Author Contributions

All authors listed have made a substantial, direct and intellectual contribution to the work, and approved it for publication.

## Conflict of Interest Statement

The authors declare that the research was conducted in the absence of any commercial or financial relationships that could be construed as a potential conflict of interest.

## Footnotes

1 ftp://toms.gsfc.nasa.gov/pub/eptoms/data/erythemal/Y2005/L3_eryln_ept_20051214.txt
